# Surgery of the Maxillary Sinus

**Published:** 1897-01

**Authors:** J. D. Patterson

**Affiliations:** Kansas City.


ARTICLE III.

### "SURGERY OF THE MAXILLARY SINUS."

BY DR. J. D. PATTERSON, KANSAS CITY.

**Read before the Interstate Dental Meeting at Excelsior Springs, Mo., June 23-26, 1896.**

On account of contiguity of the sinus to the superior molar roots, disease of these latter often extends to the antrum. Diseases of these teeth are too often treated without investigating the antrum, especially in obstinate and chronic cases. The treatment of diseases of this cavity has not been properly taught, so that the ordinary practitioner is not at all skilled in their diagnosis. The utmost skill is often demanded in making a correct diagnosis. The symptoms are often obscure and deceptive. When from a diseased tooth the diagnosis is often simple, but from other causes is more obscure. When the teeth are not affected the symptoms are often similar to, and are often mistaken for, a severe coryza. The teeth do not respond, and there is no redness or swelling. The most ordinary and evident signs are not present. Sometimes an electric lamp in the mouth will show through the antrum and cheek, but even the translucency cannot be depended upon. The new X rays may prove valuable in diagnosing diseases of this kind. When the usual symptoms are present—heat, swelling, distention, etc.—and are plain, there is sufficient ground for operating. The diseased tooth should be extracted, and this alone will often effect a cure. When chronic, however, this will not be sufficient. The antrum must then be opened, and there has been much dispute as to the proper place for performing this operation. I have found that the best place generally is the lowest level of the space formed by the triangle of three roots of the upper first molar. First sterilize your instruments in resorcin or cassia water. All instruments and surroundings must be aseptic. The engine makes such work much easier and more rapid nowadays. The trephine shown should be first used, with little pressure, as it will progress rapidly. When the cavity is reached, a flow of diseased matter will come. After this is well evacuated follow with the larger instruments until you can insert the little finger and explore all parts of the cavity and see as much of it as possible. Wash with antiseptic liquids and dress as may be necessary, with water at one hundred degrees, with a pinch of salt to allay pain, which it will do. Tumors and growths must be removed with curets. These are difficult to treat, but can be removed successfully and a cure effected (as described in a given case.) It may be safely said that even malignant growths may be removed and a cure effected if taken soon enough. Complete bony walls isolate the growth, and a perfect cure can be attained by their removal if taken before the bone has become affected. —*Cosmos.*

